# Emergence of native peptide sequences in prebiotic replication networks

**DOI:** 10.1038/s41467-017-00463-1

**Published:** 2017-09-05

**Authors:** Jayanta Nanda, Boris Rubinov, Denis Ivnitski, Rakesh Mukherjee, Elina Shtelman, Yair Motro, Yifat Miller, Nathaniel Wagner, Rivka Cohen-Luria, Gonen Ashkenasy

**Affiliations:** 1 Department of Chemistry, Ben-Gurion University of the Negev, Beer-Sheva, 84105 Israel; 2 Present Address: Department of Chemistry, Indian Institute of Engineering Science and Technology, Shibpur, Howrah, 711103 India

## Abstract

Biopolymer syntheses in living cells are perfected by an elaborate error correction machinery, which was not applicable during polymerization on early Earth. Scientists are consequently striving to identify mechanisms by which functional polymers were selected and further amplified from complex prebiotic mixtures. Here we show the instrumental role of non-enzymatic replication in the enrichment of certain product(s). To this end, we analyzed a complex web of reactions in β-sheet peptide networks, focusing on the formation of specific intermediate compounds and template-assisted replication. Remarkably, we find that the formation of several products in a mixture is not critically harmful, since efficient and selective template-assisted reactions serve as a backbone correction mechanism, namely, for keeping the concentration of the peptide containing the native backbone equal to, or even higher than, the concentrations of the other products. We suggest that these findings may shed light on molecular evolution processes that led to current biology.

## Abstract (web summary)

The synthesis of biopolymers in living cells is perfected by complex machinery, however this was not the case on early Earth. Here the authors show the role of non-enzymatic replication in the enrichment of certain products within prebiotically relevant mixtures.

## Introduction

Cells use a surprisingly small number of molecules, primarily nucleic acids, proteins, fatty acids, and sugars, to perform an immense repertoire of functions. Even more remarkable is the phenomenon that only small subsets of these molecular families, containing distinctive backbone connectivity and chiral arrangements, have evolved to be operative. For example, today’s DNA and RNA molecules contain the d form of (deoxy)ribose attached solely at the 3′–5′ positions of successive backbone units, and proteins are made up almost entirely of l type α-amino acids, connected through their α-carboxy and α-amino termini. The evolution of these unique polymer sequences—in preference to others—from a huge prebiotic configurational space remains a mystery^1–3^, since in the absence of highly evolved genetic machinery or enzyme catalysis most polymer condensation reactions result in mixtures containing a large number of products, typically isomers of the ‘‘desired’’ molecule, each produced in low yield.

**Fig. 1:**
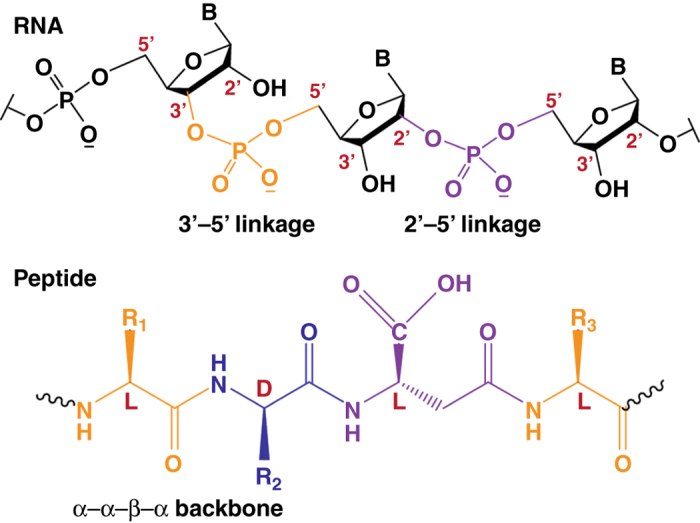
illustration of RNA and peptide molecules with heterogeneous backbones

Many experiments directed at spontaneous polymerization of biomolecules have indeed yielded mixtures containing RNA molecules with heterogeneous backbones^4–6^, or peptides with mixed chirality^7–10^, in some cases elongated at their side chains (Fig. 1). Elucidating the mechanisms by which functional prebiotic polymers were selected and further amplified may offer ways to resolve this ‘‘prebiotic chemist’s nightmare’’^3, 11, 12^. Achieving this aim may explain the early molecular evolution processes that led to biology as we know it today.

In the context of the emergence of an RNA world, researchers have suggested that RNA was preceded by transient informational oligomers that were compatible with pre-biological pathways, which, in turn, could support the transition to RNA itself^1, 13^. Based on RNA replication experiments, it has also been proposed that the difficulty presented by chemical heterogeneity to the understanding of early evolution has been exaggerated, since some chemical heterogeneity is actually tolerable in polymerization and replication processes^5, 14^. With respect to chirality, several studies have shown that even minute differences in abundance (i.e., enantiomeric excess) can be amplified when various self-assembly processes, including crystallization, occur^8, 15–20^. In addition, it has been postulated that the delivery of enantio-rich amino acids from outer space was the origin for homo-chirality^21, 22^. Active polymerization-racemization processes^18^, and differential breakdown of incorrect product(s) followed by channeling resources into the correct product^23–25^, comprise additional putative pathways for the formation of chiral peptide oligomers. Furthermore, regio-enantioselective in the steps of chain elongation by growing β-sheet peptides yielded isotactic sequences^8, 26, 27^, albeit often forming racemic aggregates. Interestingly, a recent study (with RNA) has shown that both cross-catalytic and anti-cross catalytic of one handedness or the other are tolerable^28^.

Herein, we put forward another selection mechanism relevant to early chemical evolution, since we probe the instrumental significance of non-enzymatic replication for the amplification and fixation of certain products within heterogeneous prebiotic mixtures. In particular, we demonstrate that β-sheet-driven peptide replication can be used for significant enrichment of a native condensation product in preference to some of its regio-isomers and stereo-isomers and hence for correction of the evolutionary drift towards non-functional heterogeneous mixtures.

**Fig. 2:**
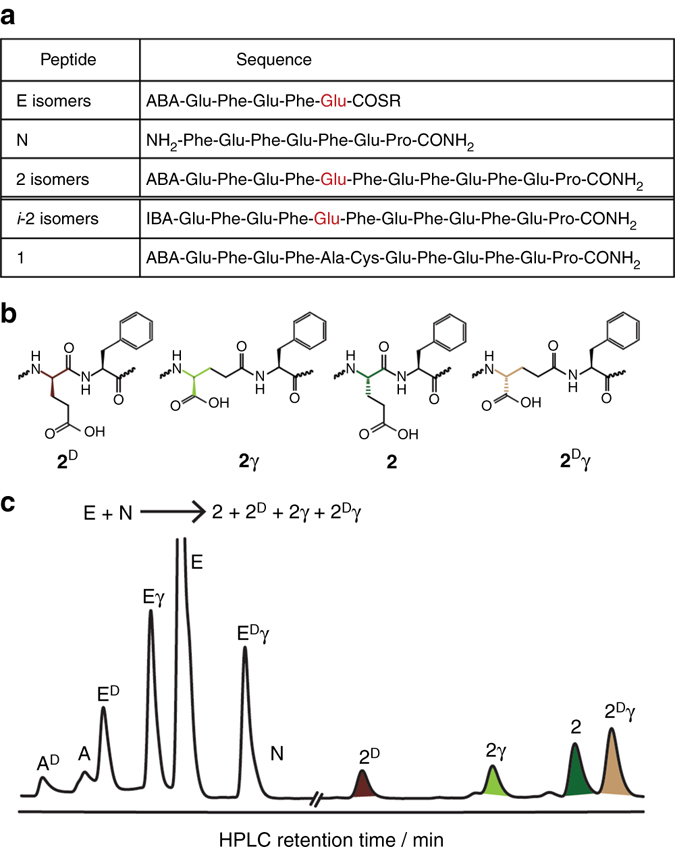
Peptides utilized for the current study. **a** Peptide names and their sequences. Sequences with a glutamic acid residue marked in *red* represent the four isomeric species discussed in the text and shown in **b**. *ABA* 4-acetamidobenzoate, *IBA* 4-*iso*-butylamide benzoate, and *SR* 4-mercaptophenylacetic acid (*MPAA*). **b** Absolute configuration and backbone connectivity of the ligation site Glu residue in **2** and its isomers **2** ^**D**^, **2γ**, and **2** ^**D**^ **γ**. **c** Representative HPLC chromatogram (detected at 270 nm) obtained during a ligation reaction initiated by 200 μM **E** and 300 μM **N**. Note that schemes detailing the ligation reaction steps are given in Fig. 5 and Supplementary Fig. 13. The chromatogram allows identification of all the species forming the reaction network, including the products **2**, **2** ^**D**^, **2γ**, and **2** ^**D**^ **γ**, the isomeric electrophile molecules (**E** ^**D**^, **Eγ**, and **E** ^**D**^ **γ**), and the intermediate anhydride species (**A** and **A** ^**D**^, Supplementary Fig. 1a). The electrophile and product isomers were all analyzed by LCMS, and their elution times were compared to those of the respective originally synthesized compounds (Supplementary Fig. 1b). **A** and **A** ^**D**^ peptides were identified by LCMS. Note that peptide **N** could not be well detected, since it lacks ABA labeling, and that two electrophile hydrolysis products, eluted at early times, are not shown (see SI Supplementary Methods)

The involvement of peptides in early evolution has been advocated several times^29–33^ and has also been suggested as a complementary or alternative route to the RNA world, leading to primitive life-like functions^34–39^. Recent studies have indeed revealed multiple assembly pathways, yielding amphiphilic peptide fibrils or nanotubes and, consequently, the emergence of new functions, such as catalysis, binding of nucleic acids and charge transfer^40–47^. Of particular relevance to studying the origin of life is the search for short oligopeptides, spontaneously formed and evolved as auto-catalysts^48–50^. Accordingly, we have previously studied the replication of a simple amphiphilic peptide (**1**, Fig. 2a) that, when forming β-sheet architectures, can efficiently accelerate its own formation from a solution containing the appropriate precursors^48, 51, 52^. The current study takes a different direction, in that here we applied original design and experimental conditions for inducing spontaneous evolution of a mixture containing isomeric compounds, each presenting a different phenotype following self-assembly. We thoroughly analyzed the complex web of reactions in this network via experiments and simulation with the aim to elucidate the role of the initial conditions (seeded precursors), the stability of the intermediate compounds yielding different products, and template-assisted replication. Remarkably, we find that when the replicating molecule is also the condensation kinetic product, modest replication exerted, but when the network reaction is initiated with materials leading primarily to other products with a non-homogenous backbone, efficient correction takes place by replication and enrichment of the native peptide at the expense of other network components. We conclude this paper with a brief discussion of some implications of our findings, highlighting plausible relevant pathways in early molecular evolution.

## Results

### Spontaneous evolution of a peptide network via glutamic acid ligation

This study focuses on the unique non-enzymatic replication that takes place in a small network made up of peptide **2** and its isomers (Fig. 2). The sequence of this peptide contains only repeating Glu–Phe residue dyads and capping groups. It is, accordingly, simpler than peptide **1** and potentially more prebiotically relevant. The main differences between the replication of **2** and that observed earlier for **1** originate from the mechanism of their precursors’ ligation. The replication of **1** was driven by the efficient and selective ‘‘native chemical ligation’’ reaction^53^, propelled by an alanine thioester electrophile and a dedicated Cys residue at the nucleophile N terminus. The ligation of **2**, in contrast, is achieved by strong activation of the electrophile **E** as a glutamic acid thioester, while the free Phe amine of nucleophile **N** remains non-activated (Fig. 2). As a result, the formation of **2** takes place together with the appearance of side products, structural isomers of **2** (Fig. 2), as was also observed previously in similar Asp-thioester- or Glu-thioester-based ligation reactions^54, 55^. The three isomers differ from **2** in terms of epimerization at the ligation site Glu residue (**2** ^**D**^ product) and/or in terms of non-canonical connectivity to the next Phe residue via the Glu γ-carbonyl (**2** ^**D**^ **γ **and **2γ**, respectively). Remarkably, we observed significant production of these isomers, which implies that the epimerization and isomerization processes are fast in comparison with the strategic ligation.

**Fig. 3:**
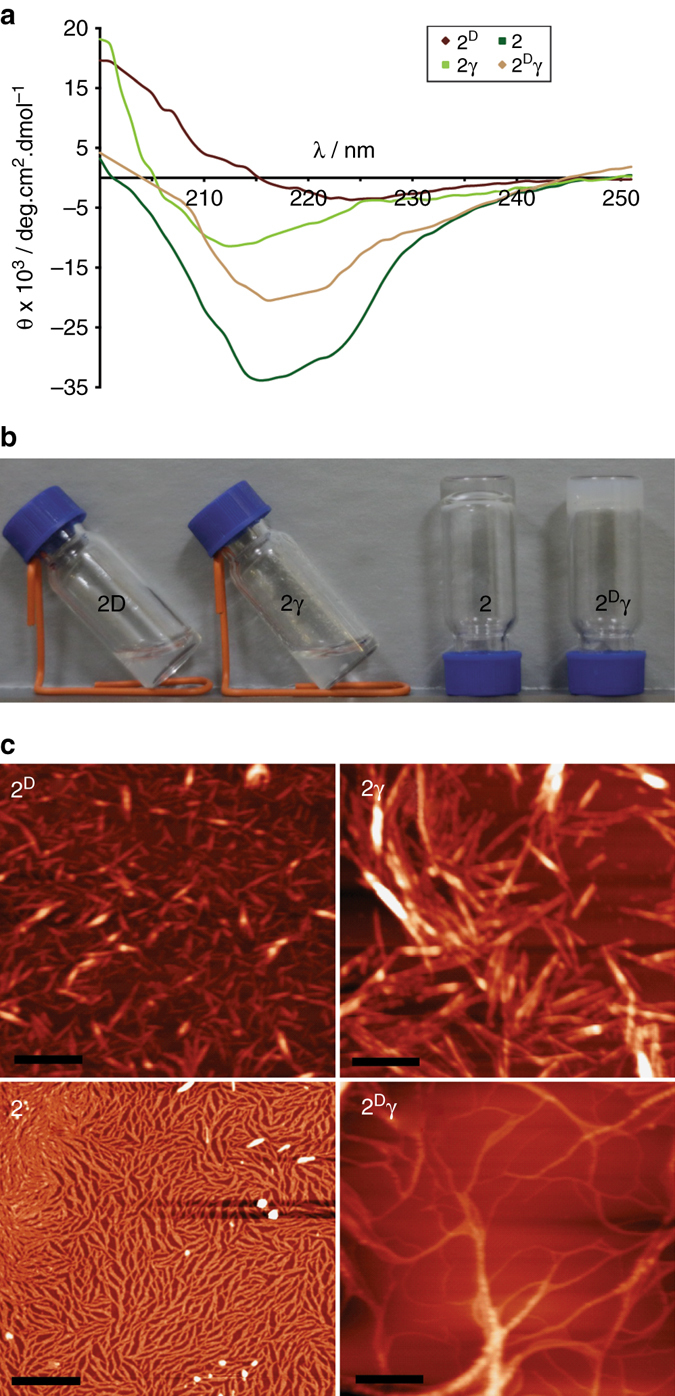
Structural characterization the four isomeric peptides **2**, **2** ^**D**^, **2γ,** and **2** ^**D**^ **γ **formed spontaneously during the ligation reaction. **a** CD spectra of 50 ± 2 µM peptides at pH 7. **b** Gelation analysis for 5.6 mM aqueous solutions at pH 7. **c** AFM images of the self-assembly products formed in 50 ± 2 μM solutions. Samples were drop cast on a mica surface after 10 min of sonication, followed by 30 min of equilibration in MOPS buffer, pH 7. All *scale bars* are 1 μm

The substantially different elution times in the high-performance liquid chromatography (HPLC) product region (Fig. 2c) mean that the hydrophobicity of peptide **2** and its three isomers was markedly different. Remarkably, further analysis using a set of common β-sheet characterization tools (Fig. 3) shows that even small structural changes next to the ligation site lead to formation of isomers (‘‘prebiotic mutants’’) exhibiting different phenotypes. Inspection of the 216-nm minima in the CD spectra of the isomers (Fig. 3a) indicates efficient assembly into β-sheets for **2**, and to a lesser extent for **2** ^**D**^ **γ**, while a practically unstructured arrangement was detected for **2** ^**D**^ and **2γ**. A qualitative gelation study yielded similar results in that, at a designated concentration, peptide **2** formed a transparent gel phase, whereas **2** ^**D**^ **γ** gelled into an opaque phase, and **2** ^**D**^ and **2γ** remained soluble (Fig. 3b). A further indication of this assembly trend was evident in atomic force microscopy (AFM) images (Fig. 3c) showing that peptide **2** formed denser fibers, and peptide **2** ^**D**^ **γ ** formed longer fibers, vs. **2** ^**D**^ and **2γ**. Since peptide **2** possesses an amphiphilic sequence and a native homogenous backbone, its strain-free assembly into β-sheets is intuitively clear. From time-dependent cryo-transmission electron microscopy (TEM) measurements (Supplementary Figs. 2 and 3), we find that the dynamic assembly of **2** proceeds via a pathway similar to that observed earlier for peptide **1** ^51^, namely, first the formation of elongated fibril structures and then, at longer times (*t* ≥ 60 min), rearrangement into hollow tubes. Our recent molecular dynamic simulations revealed that well-defined fibrils of **2** are formed by peptide bilayers in which the monomers are oriented anti-parallel to each other, both within the same layer and in opposite layers^52^. A logical explanation for the variability in assembly propensity of the other isomers may be obtained from simple modeling of their backbone orientations (Supplementary Fig. 4). Such an analysis did indeed show that native bond angles within the singly isomerized isomers **2** ^**D**^ and **2γ** would force the incorporation of the hydrophilic Glu residue side chains into the fibril hydrophobic core, with overall destabilization of the fibril structure. In contrast, the backbone of **2** ^**D**^ **γ **, isomerized twice with respect to **2**, can tolerate the assembly of stable fibers, since their hydrophobic core is occupied only by Phe residues, with the mutated Glu residue presenting its α-carboxylate toward the water phase.

### Mechanisms of the network reaction and dynamic isomerization

**Fig. 4:**
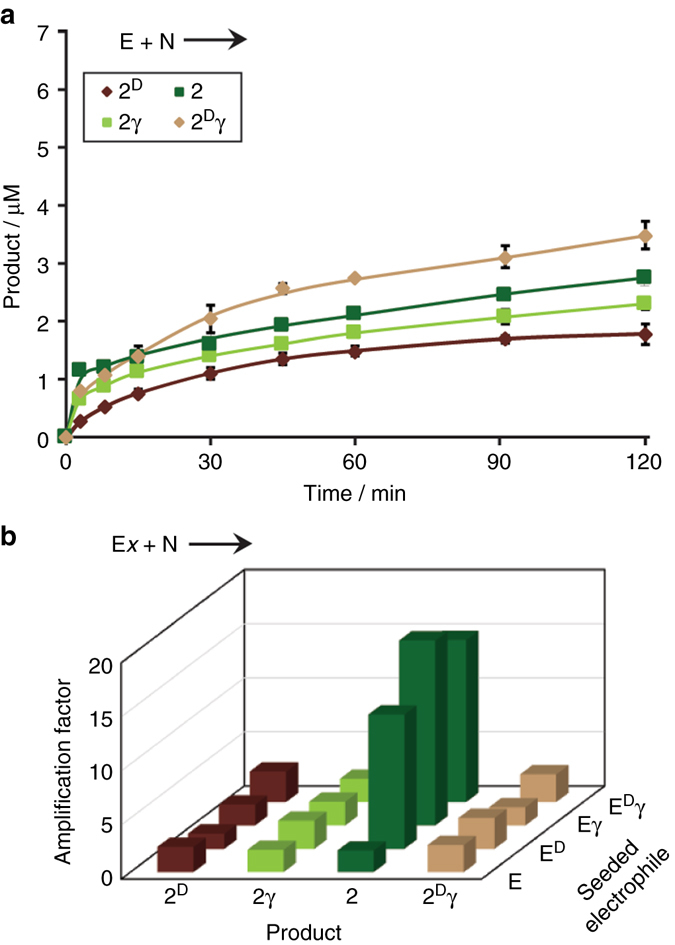
Product formation in template-free reactions. **a** Time-dependent formation of the four isomeric products in a reaction between 200 μM **E** and 300 μM **N**. **b** Amplification factors observed in the formation of the isomeric peptides in reactions initiated with **N** and each of the four electrophiles, **Ex**, separately. The amplification factors were calculated from ratios of product formation at 120 min and 15 min for reactions initiated with the α-carboxy thioesters (**E** or **E** ^**D**^; Supplementary Fig. 5), and the ratios at 240 and 90 min for reactions initiated with the γ-carboxy thioesters (**Eγ** and **E** ^**D**^ **γ**; Supplementary Fig. 5). All reactions were carried out in MOPS buffer at pH 7. Product concentrations were calculated from the HPLC chromatogram by using an ABA-labeled peptide (ABA-Ala-Ala-NH_2_) sample of known concentration as an internal standard

**Fig. 5:**
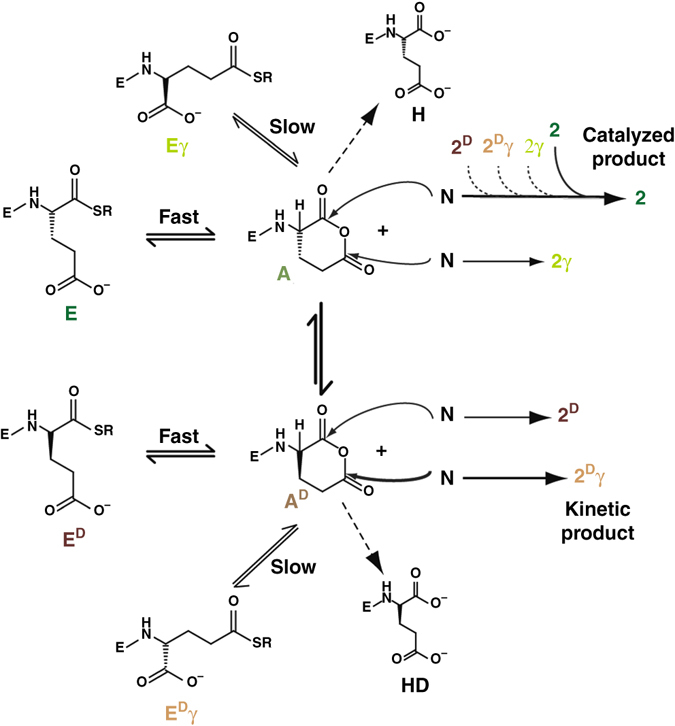
General mechanism of a network reaction initiated by **N** and any one of the electrophiles **Ex**. The scheme illustrates the central role of the glutaric anhydride intermediates **A** and **A** ^**D**^ in the dynamic exchange between the starting material and the product isomers, as well as the kinetically favored pathways leading to product **2** ^**D**^ **γ** and the catalyzed pathways leading to product **2** containing the native backbone

We then followed the kinetics of product formation during ligation between peptides **E** and **N** with the aims to expose the mechanistic features that lead to the formation of the four-isomer network and, furthermore, to explain the observed product distribution (Fig. 4a). At the early stage of the reaction (up to 15 min), the direct ligation product, **2**, formed faster than the other products. However, as the reaction progressed, **2** ^**D**^ **γ** formed efficiently and, overall, no strong preference for any one of the products was observed. The HPLC chromatograms (as in Fig. 2c) reveal that during the reaction the thioester electrophile **E** is readily converted into five reactive species, namely, three thioester isomers (**E** ^**D**^, **Eγ**, and **E** ^**D**^ **γ**) and two glutaric anhydride stereo-isomers (**A** and **A** ^**D**^; Supplementary Figs. 1a and 5e). Direct ligation of the electrophiles with **N** leads to the formation of the four products described above. We note that the two anhydrides **A** and **A** ^**D**^ constantly convert from one to the other. Consequently, these species play a central role in spontaneous mutation, as they form and epimerize very fast and can also ligate with **N**, via their α-carbonyl or γ-carbonyl carbons, to form all four products, or alternatively, react back with MPAA to form the four thioester isomers (Fig. 5).

Further insight into the network reaction mechanisms was obtained by following the time-dependent growth of the four isomeric products in reactions initiated with **N** and each of the four (pre-synthesized) electrophiles separately (Supplementary Fig. 5). The results show more efficient (total) product formation for reactions initiated with the α-carboxy thioesters **E** or **E** ^**D**^, irrespective of their end terminal Glu chirality, than for the γ-carboxy thioesters **Eγ** or **E** ^**D**^ **γ**. This observation is supported by the observed easier formation of the anhydrides (**A** and **A** ^**D**^) from the α-carboxy thioesters than from the γ-carboxy thioesters (Supplementary Figs. 5e and 6). In reactions initiated with **E** ^**D**^ or **Eγ**, similarly to the above-discussed reaction initiated with **E**, the kinetic product from direct attack on the seeded thioester formed rapidly in the early stages, but its relative abundance diminished at longer times when **2** ^**D**^ **γ** had become the most abundant product in the mixture. The putative reaction pathways leading to efficient formation of **2** ^**D**^ **γ** (Fig. 5 and Supplementary Fig. 5) were reinforced by quantitative computational analysis of the intermediate anhydride structures (Supplementary Fig. 7), which revealed that **A** ^**D**^ is more stable than **A** and that the attack on the γ-carbonyl of **A** ^**D**^ is less sterically hindered than the attack on its α-carbonyl. Most importantly, the formation of **2** was very slow in the early stages of all reactions initiated with the non-native electrophiles (**E** ^**D**^, **Eγ,** and **E** ^**D**^ **γ**), but it was significantly enhanced in later stages, with **2** reaching a final abundance similar to that of the other products (Supplementary Fig. 5). Figure 4b emphasizes that **2** was the only compound in the mixture amplified in a non-linear fashion, providing strong evidence for a error correction mechanism, via autocatalytic or cross-catalytic template-assisted replication, as further elaborated below.

### Template-directed self-replication and cross-catalytic backbone correction

The high-sequence similarity between peptide **2** and the replicator peptide **1** as well as the tendency of both molecules to form assemblies of similar β-sheet architectures strongly suggest that **2** (and/or its isomers) can also replicate. Indeed, equilibration of **2** in the presence of excess amounts of **E** and **N** peptides stabilized short-lived β-plate and fibril structures (cryo-TEM in Supplementary Fig. 3), potentially facilitating self-replication activity not hampered by transition to inactive nanotubes^51^. During a network reaction initiated by an **E** type isomer and **N**, such replication can take place either auto-catalytically, by a product formed in situ enhancing the formation of an identical molecule, or cross-catalytically with one isomer product templating the formation of another one.

**Fig. 6:**
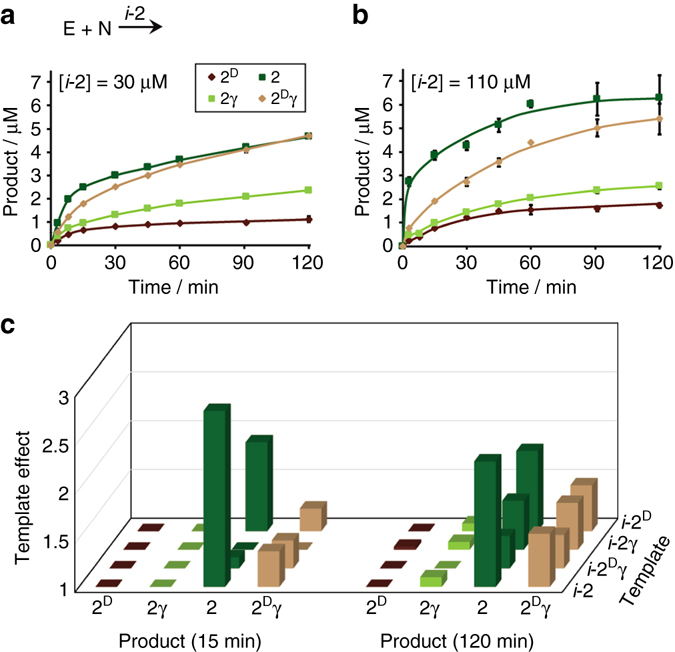
Template-assisted product formation in reactions between **E** and **N**. **a**, **b** Time-dependent product formation in reactions seeded with different *i*-**2** concentrations. **c** *Bar diagram* showing the template effect on the four products in the early (15 min) and late (120 min) stages of reactions seeded with 100 ± 10 μM of each one of the respective templates, separately. Template effect at a particular time is the ratio of product formation in a template-assisted reaction (this figure **b** and Supplementary Fig. 9) to that in the template-free reaction (Fig. 4)

The template-assisted effects in reactions initiated by **E** and **N** were investigated by seeding in the external templates *i*-**2**, *i*-**2** ^**D**^, *i*-**2γ,** or *i*-**2** ^**D**^ **γ** (Fig. 2a) very close analogs of **2**, **2** ^**D**^, **2γ,** or **2** ^**D**^ **γ**, respectively, assembled into identical fibril structures (Supplementary Fig. 8). Use of *i*-**2** as a template enhanced the initial production rate of **2** by up to threefold as compared with the template-free reaction (Fig. 6). This modest increase in rate correlated monotonically with increasing the amount of seeded template, and at high template concentrations **2** became the most abundant product in the network (Fig. 6b). A smaller increase in the production rate (~ 50%) was observed for **2** ^**D**^ **γ**, and the growth of the other two products (**2** ^**D**^ and **2γ**) remained unchanged or even slightly reduced, probably due to fast and guided consumption of the common intermediates, **A** and **A** ^**D**^, by **N**. The selective enhancement of product **2** formation by *i*-**2** may be explained by the perfect matching between the template fibrils as catalysts and the product. Seeding experiments initiated with *i*-**2** ^**D**^ **γ**, *i*-**2γ**, or *i*-**2** ^**D**^ induced an increase in rate of production of **2** (up to twofold in case of seeding with *i*-**2** ^**D**^), and lower or equal rate enhancement of the respective autocatalytic product (Fig. 6c). This observation clearly advocates for that primitive correction mechanism drives the network progress, since each of the non-native isomers (**2** ^**D**^ **γ**, **2γ**, and **2** ^**D**^) is apparently less effective as an autocatalyst than as a cross-catalyst producing the native sequence **2**.

**Fig. 7:**
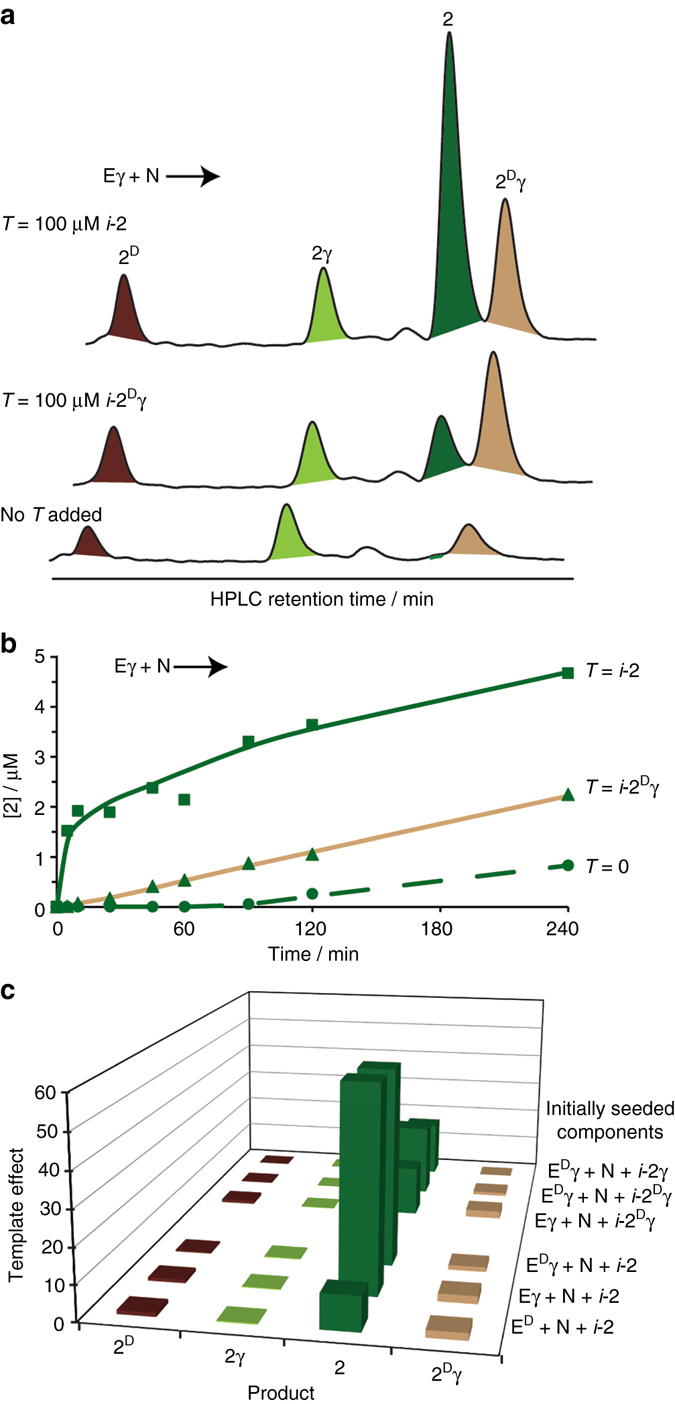
Template-assisted product formation in reactions initiated with **N** and each of the non-native electrophile thioesters (**E** ^**D**^, **Eγ**, and **E** ^**D**^ **γ**) separately. **a** HPLC chromatograms comparing product formation at 90 min in template-free and template-seeded reactions initiated with **Eγ** and **N** (seeding with *i*-**2** or *i*-**2** ^**D**^ **γ**). **b** Time-dependent formation of the native product **2** in template-free and template-seeded reactions initiated with **Eγ** and **N**. **c** *Bar diagram* representing the template effects in reactions initiated with **N** and each of the non-native electrophile thioesters, separately, and seeded each time with a different template (100 ± 10 μM). Template effects were calculated from the respective product formation data at 15 min for reactions initiated with **E** ^**D**^, and at 90 min for reactions initiated with **Eγ** or **E** ^**D**^ **γ** in template-free (Supplementary Fig. 5) and template-assisted (***b*** of this figure and Supplementary Figs. 10–12) reactions

The above-discussed template-free reactions initiated by **N** and each of the non-native thioesters, **E** ^**D**^, **Eγ**, and **E** ^**D**^ **γ** (Fig. 4b), exhibited non-linear formation of peptide **2**, strongly suggesting that efficient template-assisted processes facilitate its growth in such mixtures. This hypothesis was directly probed by performing a comprehensive set of experiments, in which the network reaction was initiated with **N** and one of the non-native thioesters in the presence of one of the four product isomer analogs (*i*-**2**, *i*-**2** ^**D**^, *i*-**2γ**, or *i*-**2** ^**D**^ **γ**; Fig. 7 and Supplementary Figs. 10–12). Marked rate enhancement (up to 60-fold) in the formation of **2** was observed in reactions seeded with *i* **-2** vs. the template-free reactions. The efficient ‘‘quasi’’ self-replication of the native peptide **2** diverted the original reaction pathway, leading to the formation of **2** as a major product in the mixture (Fig. 7a). Remarkably, the formation of **2** was selectively enhanced, while the formation rates of the three other isomers were similar, or only slightly higher, vs. those observed in the template-free reactions (up to threefold initial rate enhancement observed for **2** ^**D**^ **γ**). Interestingly, seeding in any of the other templates (*i*-**2** ^**D**^, *i*-**2γ**, or *i*-**2** ^**D**^ **γ**; Fig. 7) also resulted in rate enhancement of the production of **2**, albeit with lower efficiency, revealing a second latent mechanism for correction via cross-catalysis.

### Simulation analysis of the replication network topology

The various product distribution patterns observed in the template-free and template-seeded network reactions allow us to propose an overall network topology facilitating the synthesis of four isomeric peptides and, in particular, the amplification of product **2** possessing the native backbone structure (Fig. 5). While the most efficient template-free reaction pathways lead to formation of the **2** ^**D**^ **γ** product, template-assisted reactions—both self-replication and cross-catalysis—enhance the formation of **2**. Remarkably, as typically seen for network-level correction processes, the cross catalytic reactions, in which the non-native products (**2** ^**D**^, **2γ**, or **2** ^**D**^ **γ**) catalyze peptide **2**, were found to be more efficient than the autocatalytic reactions leading to their own formation. When the reaction is initiated with the non-native isomer electrophiles, or when the anhydride analogs of the latter are formed in situ, these efficient and selective template-assisted reactions play a role in keeping the concentration of **2** equal to, or even higher than, the concentrations of the other products. Note that a similar scenario, in which condensation reactions of pre-equilibrating starting materials and intermediates are channeled through self-replication (only) to form one major product, has been described using organic an abiotic system^56^.

**Fig. 8:**
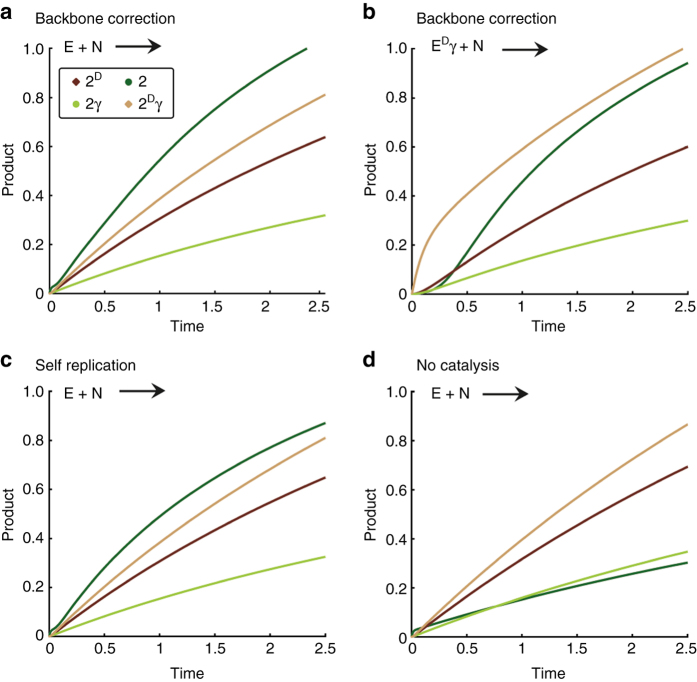
Simulation results based on the mechanism shown in Fig. 5 and the reaction model given in Supplementary Fig. 13. Experimental initial concentrations were assigned for **Ex** and **N** (250 μM each), and reaction constants were applied as specified in the SI. **a**, **b** Backbone correction growth mechanism observed when the reactions were initialized with **E** (**a**) or **E** ^**D**^ **γ** (**b**). **c**, **d** Alternate growth behavior observed when the network kinetics were governed by different sets of interactions, e.g., with more dominant self-replication **c**, or with no templated assisted processes **d**

Based on the kinetic model in Fig. 5, we ran a simulation in Matlab, using the set of equations shown in Supplementary Fig. 13. This network analysis makes use of and expands on the model we previously applied in studying the fibril-dependent self-replication of peptide **1** ^51^. Accordingly, multiple fibril growth and assembly steps (Eqs. 1–3), and template free (Eqs. 7) and template assisted (Eqs. 8) ligation reactions, account here for the formation of the four template/product isomers, including their cross reactivity when one template facilitates the replication of another one. In addition, since the new system proceeds via formation of the glutaric anhydride analogs (**A** and **A** ^**D**^, Fig. 5), the relevant steps producing, isomerizing, and consuming these anhydrides were introduced as well (Eqs. 4–6). The initial concentrations applied for the simulation directly match the concentrations we used in the experiments, while the assigned kinetic constants were grouped together to simplify the analysis when possible, as explained in the SI (Supplementary Methods). The results of this simple modeling (Fig. 8 and Supplementary Fig. 14) can remarkably reproduce many of the network kinetic features we observed in the experiments. Fig. 8a, b, and Supplementary Fig. 14a, describe the network growth profile when initiated with **N** and either one of the electrophiles **Ex**, under the conditions we defined as “backbone correction”, emphasizing that both self-replication of **2** and cross catalytic production of **2** by the other isomers take place. As was found in the above-described experiments (Fig. 4), the native product **2** formed relatively fast right from the beginning when the reaction started with **E** and **N** (Fig. 8a), while it was formed very slowly in the early stages of reactions that started with **N** and one of the other isomer electrophiles (**E** ^**D**^, **Eγ**, and **E** ^**D**^ **γ**) and only later on sped up due to efficient replication processes (e.g., Fig. 8b). The fast production of **2** ^**D**^ **γ**, due mainly to template-free processes, was also observed in these cases. The simulation can also nicely account for the observed network growth profiles under the same correction reaction conditions when seeded with the various products as templates (Supplementary Fig. 14, panels c–f). Most importantly, we have also utilized the simulation to screen for the network growth kinetics had they been governed by a different set of interactions, e.g., with much more dominant self-replication of **2** (Fig. 8c), or with no template assisted processes taking place at all (Fig. 8d). We find that very efficient self-replication of **2** may also lead to its formation as the most abundant compound, but the observed preference over production of the other isomers is lower. When no template-assisted processes take place, the kinetic product **2** ^**D**^ **γ **is the most abundant while **2** is formed very slowly.

## Discussion

The common practice of sequence-specific oligomer synthesis in organic chemistry makes use of very selective coupling reactions and the incorporation of protecting groups into reactive side chains. DNA polymerization in biology is, however, controlled by a different mechanism, namely post replication error-correction, proceeding by removal of mis-incorporated units and their replacement for the correct ones. Ill-defined protein sequences are also doomed by the cell machinery to post-synthesis digestion, enriching the population of functional proteins. Both the synthetic and biological strategies were obviously not applicable to polymer synthesis on early Earth. Scientists were thus looking for other universal mechanisms that could support sequence specific oligomer synthesis, and were accordingly attracted by the idea of post synthesis correction mechanisms. Nevertheless, in the absence of efficient catalysis by nucleases or peptidases, the reversible processes needed for breaking the nucleic acid or peptide backbone, and for recycling their fragments as building blocks in correct synthesis, are very slow. For that matter, dynamic covalent chemistry (DCC) was developed recently, enabling continuous equilibration of building blocks and products, thus leading to selection of the most fit compound(s) in the mixture, depending on the applied environmental conditions, templating molecules and sometimes even function^57, 58^. However, as of now, DCC cannot support reversible chemistry of native peptide bonds and is consequently only partially useful for studying prebiotic protein evolution. We have consequently searched for another systems chemistry approach^59^, whereby the enrichment of one ‘‘master sequence’’ is a result of a collective network behavior manifested by multiple template-assisted processes. Similar to previously reported results with coiled-coil peptide networks^38^, the synthesis of a native (or wild-type) peptide in our system is significantly enhanced in a small isomer population. Our results hint at an alternative backbone correction mechanism that could have developed within simple chemical networks through the emergence of shorter β-sheet polymers with efficient and selective catalytic behavior. In such a scenario, the formation of several products in a mixture is not critically harmful, since this is accompanied by a slow progression of the production of one molecule at the expense of the others, leading finally to the formation of a dominant functional polymer backbone. In the future, we wish to study the relevance of our approach to replication systems facilitating the condensation of more than two peptide units, and ultimately even the sequence specific addition of amino acids. Naturally, the success in developing such system will depend on further improvement in selectivity toward the desired sequence. Finally, we note that our results are in line with the old notion that the emergence of a primitive replicator was crucial and potentially sufficient for the origin of life further down the road^60^.

### Data availability

The data that support the findings of this study are available from the corresponding author upon request.

## Electronic supplementary material

Supplementary Information
