# Supplementary material for: Emergence of native peptide sequences in prebiotic replication networks
Source: Nat Commun. 2017 Sep 5;8:434. doi: 10.1038/s41467-017-00463-1 (PMC5585222; doi:10.1038/s41467-017-00463-1)
Supplement: Supplementary file 1 — Supplementary Information [file 41467_2017_463_MOESM1_ESM.pdf]

Description of Supplementary Files

File Name: Supplementary Information
Description: Supplementary Figures, Supplementary Table, Supplementary Methods and Supplementary References

Supplementary Methods

General

Chemicals and reagents were purchased from Aldrich or Merck and unless otherwise specified used without further purification. Amino acids, resins and coupling reagents were purchased from Novabiochem and Alfa Assar. DMF was purchased from biotech grade. Analytical HPLC was performed on a Dionex 1100 using a reverse phase C18 column at a flow rate of 1.5 mL/min. Preparative HPLC was performed on a Dionex Ultimate 3000 instrument using a C18 reverse phase preparative column at a flow rate of 20 mL/min. Mass spectrometry analysis was performed by LCMS Thermo Surveyor 355.

Synthesis

Synthesis of peptide templates and nucleophile. Replicator peptide **2** and its analogues (**1,** $\mathbf{2^D}$, **2**γ, $\mathbf{2^D}$γ, **i-2**, **i-**$\mathbf{2^D}$, **i-2**γ, **i-**$\mathbf{2^D}$γ and $\mathbf{2_{F6C}}$), and the nucleophile **N**, were synthesized on solid phase using Rink-Amide resin using Fmoc based chemistry with HBTU as the coupling agent. For better detection in HPLC, all the peptides, except **N**, were tagged with the chromophore acetamido-benzoic acid (ABA) having a strong absorption at 270 nm. Peptides were cleaved off the resin using trifluoroacetic acid (TFA) mixture with the proper scavengers, and then purified by preparative RP-HPLC, with a step gradient of solvents A (0.1 M NH_4HCO_3 in water; pH = 8) and B (acetonitrile). The identity and purity of the peptides were then analyzed by analytical HPLC and LCMS.

Synthesis of electrophile peptides. All electrophilic peptides (**E**, $\mathbf{E^D}$, **E**γ, and $\mathbf{E^D}$γ) were synthesized on solid phase using Dawson Dbz AM resin using Fmoc based chemistry with HBTU as the coupling agent. N-terminally Fmoc protected glutamic acid with the respective chirality and orthogonally protected (-OBz) on the relevant carboxylic acid was introduced into the resin as the first amino acid. After completing the electrophile sequence synthesis and cyclization producing the N-acyl (Nbz) derivative, the peptides were cleaved off the resin using TFA and the proper scavengers. The Glu-attached leaving group (Nbz) was readily exchanged for an aromatic thiol, 4-mercapto phenyl acetic acid (MPAA), and the orthogonal (-OBz) protection was removed from the first glutamic by a mixture of TFA/trimethylsilyl trifluoromethane sulfonate/cresol (10:2:2). All the electrophiles were purified by preparative RP-HPLC, with a step gradient of solvents A (99 % water, 1 % acetonitrile and 0.1 % TFA) and B (90 % acetonitrile, 10 % water and 0.07 % TFA). The identity and purity of the peptides were then analyzed by analytical HPLC and LCMS.

Peptide fibril structural characterization

Preparation of stock solution for the biophysical studies. Stock solutions of about 500 µM peptides (**2**, **2^D**, **2**γ and **2^D**γ) used for all structural characterizations (CD, AFM and cryo-TEM) and kinetic experiments, were prepared by weighing lyophilized peptides into Eppendorf tubes, dissolution in Millipore water/ACN (1:1), and then sonication (at 40 kHz) for 10 min. These peptide mixtures were then further diluted in MOPS buffer at pH = 7 to achieve the desired concentration. The exact final concentration of each peptide solution was determined from its UV absorbance at 270 nm, based on the extinction coefficient of ABA. The diluted solutions were also sonicated for 10 minutes in order to achieve full disassembly of peptide aggregates to the monomers, before the experiments were initiated.

Circular Dichroism Measurements. CD spectra collected for sonicated peptide solutions of 50±2 µM at 25 °C. Measurements were taken on a JASCO-815 CD spectropolarimeter, using a quartz cell with a 1.0 mm path length and 4 s averaging times. The CD signals resulting from buffer alone were subtracted from the spectrum of each peptide solution.

Cryo-TEM Imaging. Dynamic morphologies of native peptide (**2**) were studied alone and in presence of electrophile and nucleophiles in MOPS buffer (pH 7) using cryo-TEM imaging techniques. Samples for direct imaging of the aqueous dispersions were prepared in the controlled environment box (T = °C) of a vitrification robot (Vitrobot, FEI), as follows: a 5 µL drop of the solution was deposited on a glow-discharged TEM grid (300-mesh Cu lacey substrate; Ted Pella, Ltd.). The excess liquid was automatically blotted with a filter paper, and the specimen was rapidly plunged into liquid ethane and transferred to liquid nitrogen, where it was kept until use. The samples were examined below 175 °C using an FEI Tecnai 12 G^2 TWIN TEM that operated at 120 kV in low-dose mode and with a few micrometers under focus to increase the phase contrast. The images were recorded with a Gatan charge coupled device camera (model 794) and analyzed by Digital Micrograph software, version 3.1.

Atomic Force Microscopy Measurements. The self-assembly products of the four isomeric peptides (**2**, **2^D**, **2**γ and **2^D**γ) were analyzed using atomic force microscopy (AFM; SolverPro, NTMDT, Ru). To that aim, silicon substrates with native oxide layer were cleaned using a freshly prepared piranha solution (solution of 3:7 30 % H_2O_2 and concentrated H_2SO_4) for 30 minutes, followed by three immersions in Millipore water for 10 minutes (Caution: Piranha is a very strong oxidant and reacts violently with many organic materials). A peptide solution (50 µM in buffer pH 7) was introduced onto the surface (10 µL for 10 s), followed by drying under a nitrogen flow. AFM topography and phase images were acquired using noncontact tips (NSG03 NT-MDT, Ru (5.1 N m^{-1}, 150 kHz, and 1.74 N m^{-1}, 90 kHz, respectively)).

Gelation Studies. Gelation capability of the four isomeric peptides (**2**, **2^D**, **2**γ and **2^D**γ) was studied in phosphate buffer (50 mM) at pH = 7.0. Lyophilized peptide samples were added into separate screw-

capped glass vials (diameter 2 mm), and 200 μL phosphate buffer solution was added to each of the vials (fielding 5.6 mM solutions). These solutions were heated carefully on a hot-plate to dissolve the peptides until clear solutions reached. The clear solutions then allowed cooling down at room temperature for 10 minutes. Gelation was qualitatively probed and confirmed by test-tube inversion method.

Kinetic experiments

A stock solution of peptide **N** at ca. 2 mM was prepared by dissolving it into aqueous buffer (water: MOPS buffer = 4:1), and stock solution of the different isomeric **E** type electrophiles were prepared by dissolving the respective peptide into 1:1 water and acetonitrile mixtures at ~1 mM. We have confirmed that within the examined time and under these conditions (pH ~ 4.5) an electrophile peptide cannot react to form the anhydrides or the other isomers. Stock solution of the different isomeric templates (**i-2**, **i-2$^{\mathbf{D}}$**, **i-2**γ, or **i-2$^{\mathbf{D}}$**γ) were prepared by dissolving the respective peptide into 1:1 water and acetonitrile mixtures at ~0.5 mM. All the stock solutions were sonicated for 1 minute before adding to the reaction mixture. Experiments were initiated very promptly (typically within less than 30 seconds) by the addition of an electrophile peptide (**E**, **E$^{\mathbf{D}}$**, **E**γ, or **E$^{\mathbf{D}}$**γ; 200 μM) into a buffered mixture (MOPS pH 7, 200 mM) containing the nucleophile **N** (300 μM) and a designated amount of the template (**i-2**, **i-2$^{\mathbf{D}}$**, **i-2**γ, or **i-2$^{\mathbf{D}}$**γ). The progress of the reactions was monitored by taking aliquots at different time points. These were immediately quenched with 5 % trifluoroacetic acid (TFA), and stored frozen until analyzed by RP-HPLC. The concentration of each compound, reactants or product, was calculated by comparison with the HPLC peak of a foreign ABA-labelled peptide (ABA-Ala-Ala-NH_2) with a known concentration. Example HPLC chromatograms are given in Figures 2 and 7 of the manuscript, and in Supplementary Figure 1. The electrophile hydrolysed products (**H** and **H$^{\mathbf{D}}$**) peaks are not shown in Figure 2 of the manuscript; by following the rate of their appearance, we account for ≤ 25% mol equivalent hydrolysis of the electrophiles over the 2-hour experiment time.

Structure analysis by computational methods

The experimental measurements suggested two peptide isomers **A** and **A$^{\mathbf{D}}$** (Supplementary Table 1: ABA-Glu-Phe-Glu-Phe-Anhydride) that possess the L and D configurations, respectively. Moreover, the experimental measurements suggest that there is a rapid exchange between these two isomers. In order to provide insight into the energy difference between these two isomers, ab initio quantum calculations were applied.

Force-field potentials, such as AMBER, OPLS and MMFF94 are the most extensively applied tool for investigating large biological molecules. Although these force-fields are easily used and computationally fast, their validity for estimating the minimized energy of conformations of peptides

is not adequate. On the other hand, ab initio potentials can be computationally demanding and therefore may be applicable only for relatively small biological systems, such as peptides. Since calculations of the full-length anhydride peptides (ABA-Glu-Phe-Glu-Phe-Anhydride) using quantum calculations are extremely expensive, we computed the truncated analogue D and L forms of these anhydrides (Ac-Phe-Anhydride), assuming minimal effect of the rest of the peptide chain on the occupied conformations. The two truncated D and L forms were optimized using the Restricted Hartree-Fock (RHF) electronic structure with the DZV basis set in the GAMESS package.[s1,s2]

Modelling the simultaneous self-assembly and replication reactions.

Our computations were performed by simulating, in *MATLAB*, the kinetics of the chemical reactions listed in Supplementary Figure 13. The run procedure was as follows: at incremental time steps, we calculated the extent of each reaction, i.e., the product formed for the assigned rate constants and seeded reactants. After these were all calculated, the concentrations of all reactants and products were appropriately adjusted. This was repeated at each time step until the maximum time was reached. Mathematically, this is equivalent to solving the differential equations using the Euler method.

The following default rate constants were used:
$a^{\alpha L}$ = 200000; $a^{\alpha D}$ = 1000; $a^{\gamma L}$ = 1000; $a^{\gamma D}$ = 10000; a_{-1} = 0.01; accounting for the observed order in formation of stable fibrils, 2 > $2^D\gamma > 2^D \sim 2\gamma$.
d = 0.01; w = 0.01.
k^α = 0.01; k^γ = 0.001; k_{-1} = 0.000; g^α = 0.02; g^γ = 0.025; g = 0.1; accounting for the faster reactions when the system was initiated with the α-type electrophiles.
e = 0.05; e_{-1} = 0.02. reflecting the more stable structure of the D-type anhydride.
$b^{\alpha L}$ = 10000000; $b^{\alpha D}$ = 10000; $b^{\gamma L}$ = 10000; $b^{\gamma D}$ = 100000; accounting for the observed order of self-replication, 2 > $2^D\gamma > 2^D \sim 2\gamma$.
$c^{\alpha L}$ = 1000000; $c^{\alpha D}$ = 10000; $c^{\gamma L}$ = 10000; $c^{\gamma D}$ = 10000. Emphasizing the error-correction process when fibrils made of the non-native peptides, $2^D\gamma$, 2^D, 2γ, can better catalyse the growth of peptide 2 than their own replication.
These values were used to run all scenarios accounting for "Error correction", namely to produce all the graphs in the manuscript Figure 8 and the Supplementary Figure 14, except for Figures 8c and 8d in the manuscript. For the "Self-replication" case (Fig. 8c) we have applied $c^{\alpha L} = c^{\alpha D} = c^{\gamma L} = c^{\gamma D}$ = 10000, and for the "no catalysis" case (Fig. 8d) $b^{\alpha L} = b^{\alpha D} = b^{\gamma L} = b^{\gamma D} = c^{\alpha L} = c^{\alpha D} = c^{\gamma L} = c^{\gamma D}$ = 10000.
For all runs we used critical fibril sizes m = 10 and n = 100.

Using the designated parameters, we ran four background reactions initiated with Ex and N (Figs. 8a, 8b, Supplementary Figures 14a, s14b), and another four representative cases for template-seeded reactions (Supplementary Figures 14c-f). The following initial conditions were applied in all cases:

250 μM of the seeded electrophile Ex, 250 μM of the nucleophile N, S = 0, and 5 μM of the appropriate template for the template-seeded cases.

Supplementary table 1. Peptide names, sequence and measured molecular weight

Peptide	Sequence [a]	MW (calculated)	MW (measured)
**2**	ABA-Glu-Phe-Glu-Phe-Glu-Phe-Glu-Phe-Glu-Phe-Glu-Pro-CONH$_2$	1785.8	1786.7
**2^D**	ABA-Glu-Phe-Glu-Phe-(D)Glu-Phe-Glu-Phe-Glu-Phe-Glu-Pro-CONH$_2$	1785.8	1786.6
**2γ**	ABA-Glu-Phe-Glu-Phe-(γ)Glu-Phe-Glu-Phe-Glu-Phe-Glu-Pro-CONH$_2$	1785.8	1786.6
**2^Dγ**	ABA-Glu-Phe-Glu-Phe-(γD)Glu-Phe-Glu-Phe-Glu-Phe-Glu-Pro-CONH$_2$	1785.8	1786.6
**E**	ABA-Glu-Phe-Glu-Phe-Glu-CO-SR	1011.0	1011.1
**E^D**	ABA-Glu-Phe-Glu-Phe-(D)Glu-CO-SR	1011.0	1011.1
**Eγ**	ABA-Glu-Phe-Glu-Phe-(γ)Glu-CO-SR	1011.0	1011.1
**E^Dγ**	ABA-Glu-Phe-Glu-Phe-(γD)Glu-CO-SR	1011.0	1011.1
**A**	ABA-Glu-Phe-Glu-Phe-(L)Glutaric anhydride	842.8	843.3[b]
**A^D**	ABA-Glu-Phe-Glu-Phe-(D)Glutaric anhydride	842.8	843.2[b]
**N**	NH$_2$-Phe-Glu-Phe-Glu-Phe-Glu-Pro-CONH$_2$	943.0	943.4
**i-2**	IBA-Glu-Phe-Glu-Phe-Glu-Phe-Glu-Phe-Glu-Phe-Glu-Pro-CONH$_2$	1813.9	1814.4
**i-2^D**	IBA-Glu-Phe-Glu-Phe-(D)Glu-Phe-Glu-Phe-Glu-Phe-Glu-Pro-CONH$_2$	1813.9	1814.7
**i-2γ**	IBA-Glu-Phe-Glu-Phe-(γ)Glu-Phe-Glu-Phe-Glu-Phe-Glu-Pro-CONH$_2$	1813.9	1814.6
**i-2^Dγ**	IBA-Glu-Phe-Glu-Phe-(γD)Glu-Phe-Glu-Phe-Glu-Phe-Glu-Pro-CONH$_2$	1813.9	1814.4
**1**	ABA-Glu-Phe-Glu-Phe-Ala-Cys-Glu-Phe-Glu-Phe-Glu-Pro-CONH$_2$	1683.7	1683.2
**2_{F6C}**	ABA-Glu-Phe-Glu-Phe-Glu-Cys-Glu-Phe-Glu-Phe-Glu-Pro-CONH$_2$	1741.8	1741.7

[a]ABA = 4-acetamidobenzoate, IBA = 4-iso-butylamide benzoate, and SR = 4-mercaptophenylacetic acid (MPAA). [b]The anhydride intermediates were isolated in the kinetic studies performed at pH 7, quenched in 5% TFA solutions and quickly characterized by LCMS (Supplementary Figure 1a).

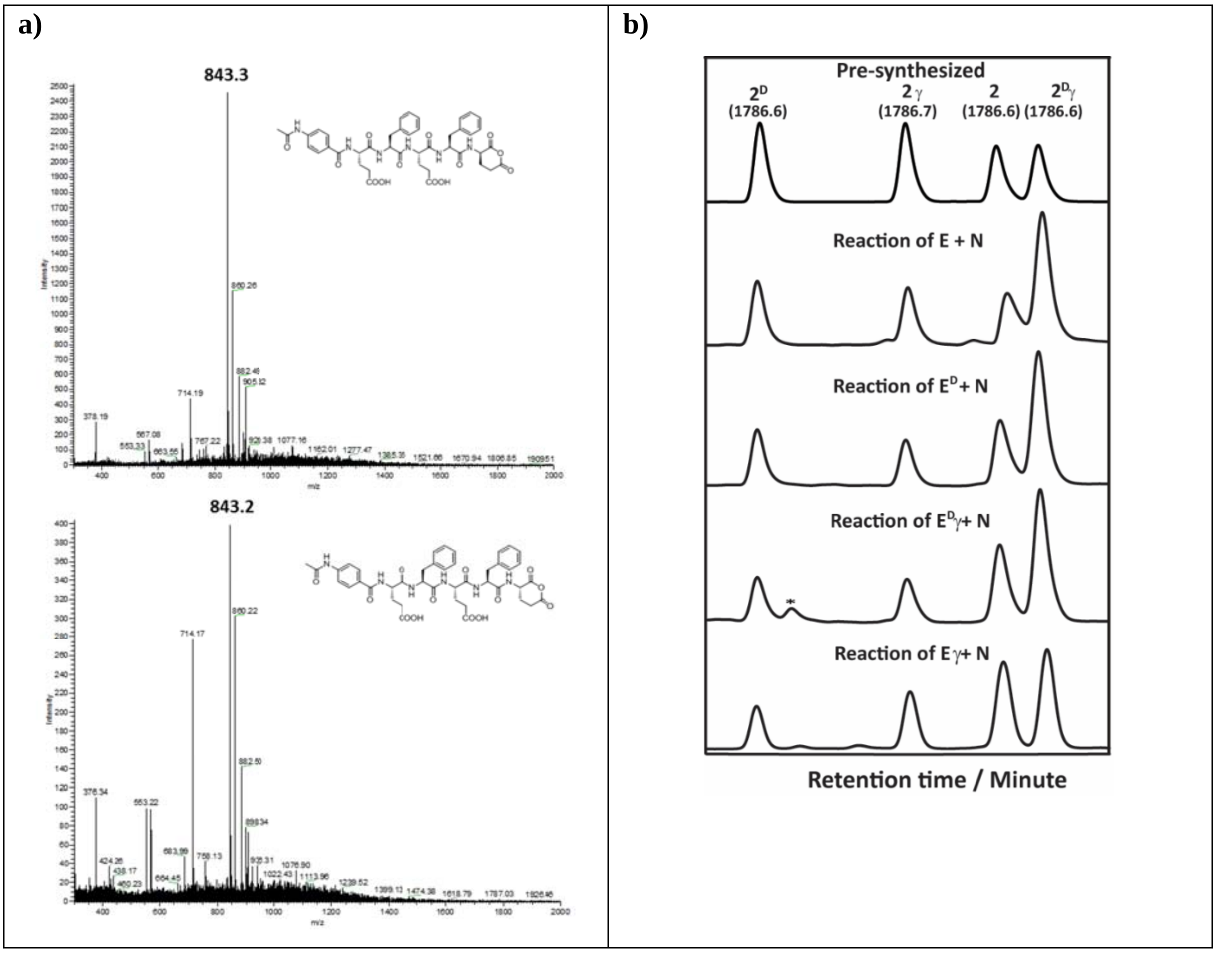

Supplementary figure 1. a) ESI-MS record of the two intermediate glutaric anhydride analogs formed in the network reaction **A** and $\mathbf{A^{D}}$. b) Representative HPLC traces of the network reaction product compounds, **2**, $\mathbf{2^{D}}$, **2**γ and $\mathbf{2^{D}}$γ. The *top* panel obtained for mixture of the original pre-synthesized peptides; values in parenthesis are the measured ESI MS of the respective eluted compound. The four other panels emphasize the product region in reactions initiated with the nucleophile **N** and each of the four **Ex** electrophiles, separately.

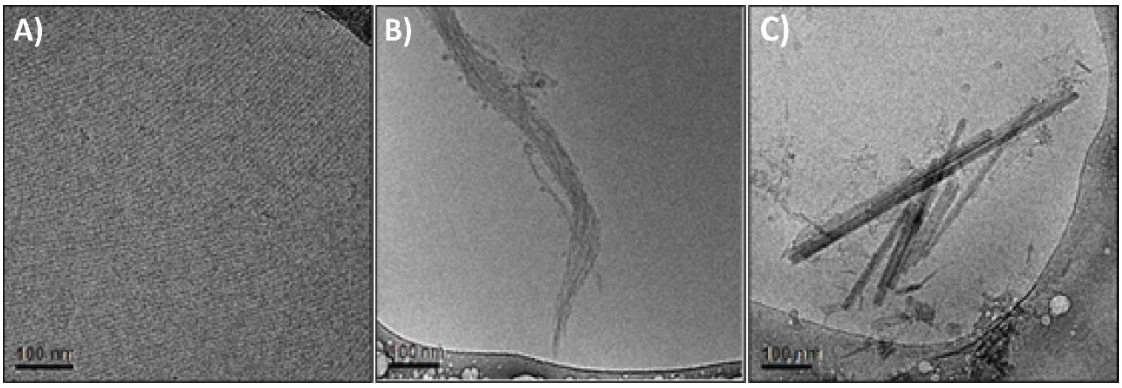

Supplementary figure 2. Cryo-TEM micrographs of vitrified structures formed in 50 µM solutions of **2** in MOPS buffer at pH 7, obtained (A) 15 min (B) 30 min (C) 60 min after sonication. Scale bars are for 100 nm. These images emphasize that the dynamic morphological transition of peptide **2** passes through β-plated sheet (A), to nanofibers (B) and then to stable nanotubes (C).

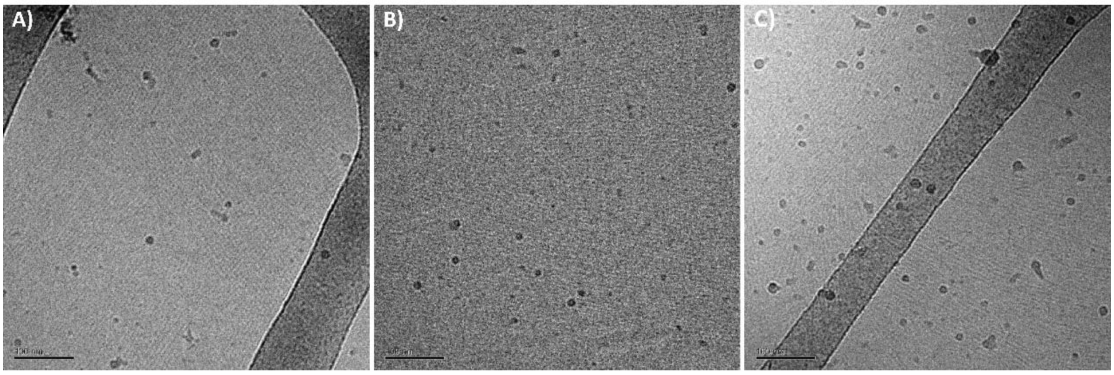

Supplementary figure 3. Cryo-TEM micrographs of vitrified structures formed in 50 µM solutions of **2** in presence of electrophile **E** (250 µM) and nucleophile **N** (250 µM) in MOPS buffer at pH 7, obtained (a) 15 min (b) 30 min (c) 60 min after sonication. Scale bars are for 100 nm. These three micrographs suggested that transient as well as the most catalytic morphology (β-plated sheet) of peptide 2 preserved in presence of electrophile and nucleophiles.

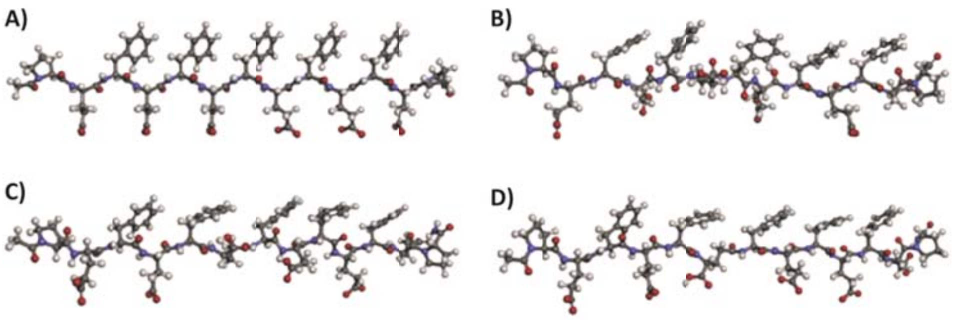

Supplementary figure 4. Balls and sticks representation of the monomeric peptide backbone as found after minimization by the CHARM27 force field for A) **2**, B) $\mathbf{2^D}$, C) **2**γ and D) $\mathbf{2^D}$γ. Peptide **2** possesses an amphiphilic sequence and a native homogenous backbone; its strain-free assembly into β-sheets is clearly observed (A). The relaxed bond angles within the singly isomerized mutants $\mathbf{2^D}$ and **2**γ (B, C) would force the incorporation of the hydrophilic Glu residue side chains into the fibril hydrophobic core, and overall destabilizing the fibril structure. The backbone of $\mathbf{2^D}$γ , isomerized twice with respect to **2** (D), can tolerate the assembly of stable fibers, since the hydrophobic core is only occupied by Phe residues, while the mutated Glu residue presenting its α-carboxylate towards the water phase.

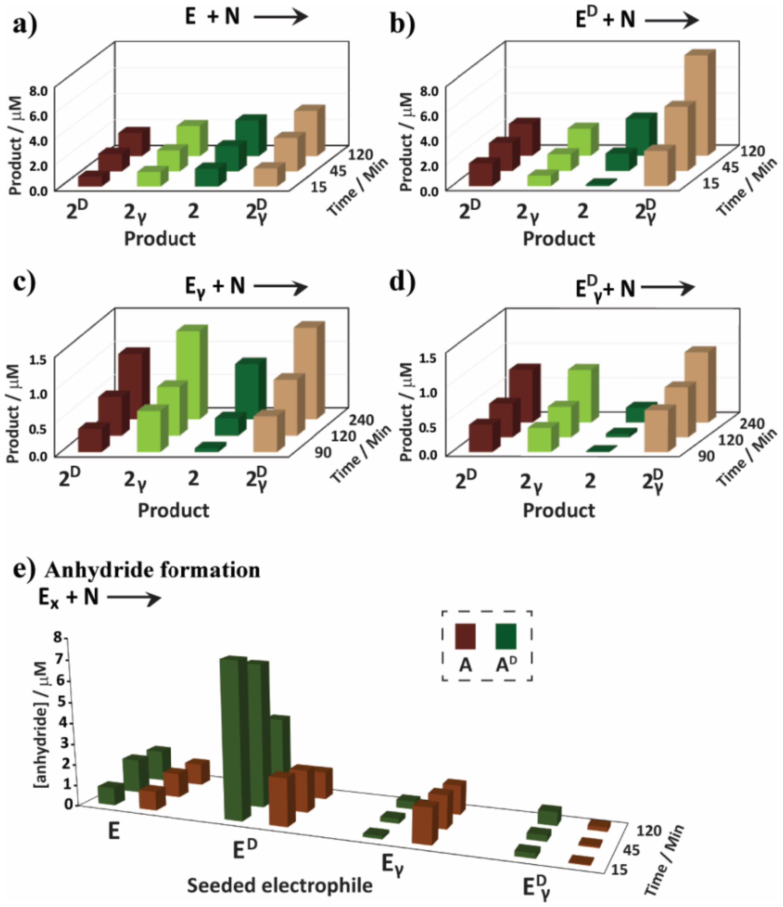

Supplementary figure 5. a – d) Bar diagrams representing the time dependent formation of the four isomeric products in template-free ligation reactions initiated with **N** and each of the four isomeric electrophiles **Ex**, separately. e) Time dependent formation of the anhydride intermediates (**A**, $\mathbf{A^D}$) along these template-free reactions.

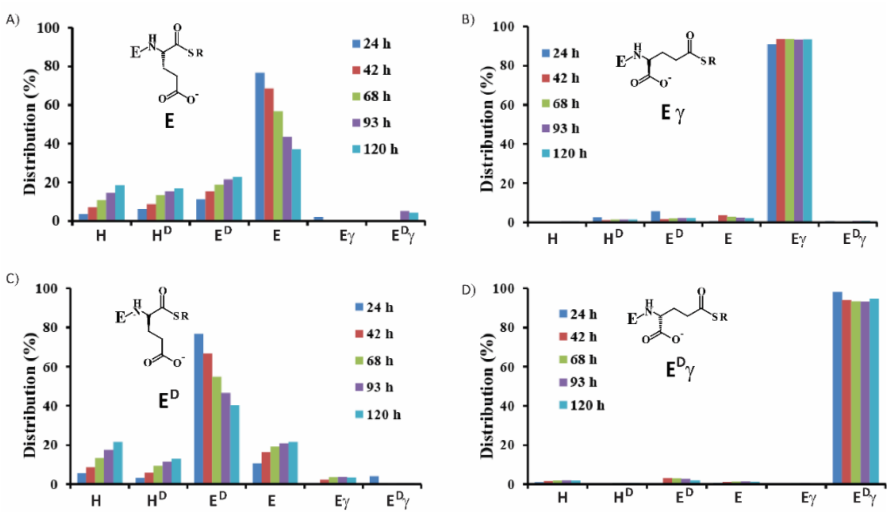

Supplementary figure 6. Dynamic exchange initiated by equilibration of each of the four **Ex** electrophiles, separately. Individual panels show the equilibration processes starting with **E** (A), **Eγ** (B), $\mathbf{E^D}$ (C) and $\mathbf{E^D\gamma}$ (D). Reactions carried out using 75±10 μM of the studied electrophile in 200 mM MOPS buffer (pH = 7) at room temperature. It is clearly observed that equilibration of the α-carboxy thioesters (**E** or $\mathbf{E^D}$) leads to much faster formation of the isomeric electrophiles, in comparison to equilibration of the γ-carboxy electrophiles (**Eγ** and $\mathbf{E^D\gamma}$). H and H^D represent the hydrolysis products with the corresponding L and D configurations. Mercaptoethane sulfonic acid (MESNa) was used as the thiol leaving group (SR) in this study.

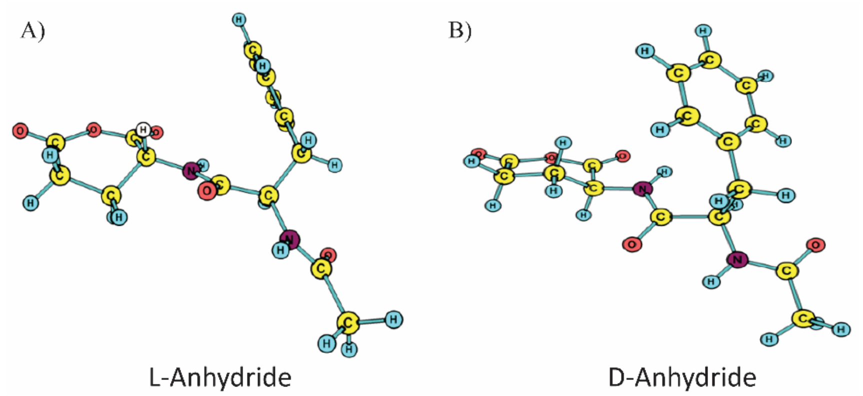

Supplementary figure 7. Energy minimized structures of the **A** (A) and $\mathbf{A^D}$ (B) anhydride analogues. In order to simplify the computational analysis, we have studied the truncated form of these anhydrides (Ac-Phe-Anhydride) instead of the full length anhydride (ABA-Glu-Phe-Glu-Phe-Anhydride). In the most stable conformation, the L-anhydride is found to be of 0.35 Kcal / mol higher in energy than the D-anhydride. The figure further allows observing that the α-carboxy carbonyl is more sterically hindered by the Phe phenyl ring, than the γ-carboxy, making the latter more accessible during ligation with the nucleophile **N**.

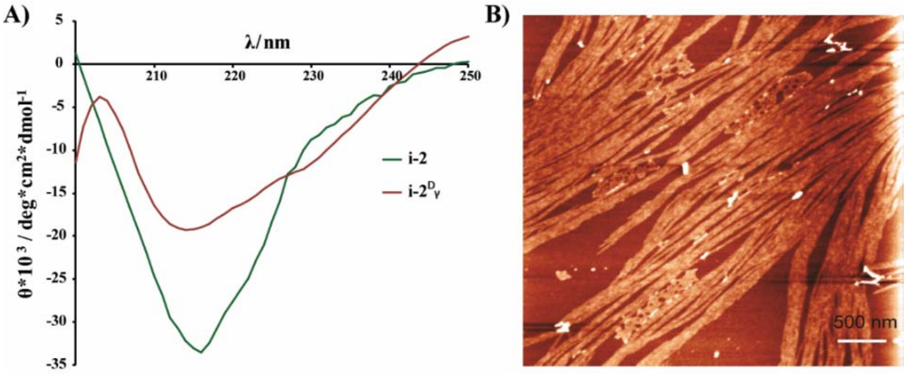

Supplementary figure 8. A) CD spectra of **i-2 and i-2^{D}**γ (50 ± 2 μM) in MOPS buffer pH 7. B) AFM image of **i-2** equilibrated in MOPS buffer pH 7. CD spectra and AFM image suggest the self-assembling nature of isomeric templates (**i-2** and **i-2^{D}**γ) is similar to the isomeric peptides (**2** and **i-2^{D}**γ).

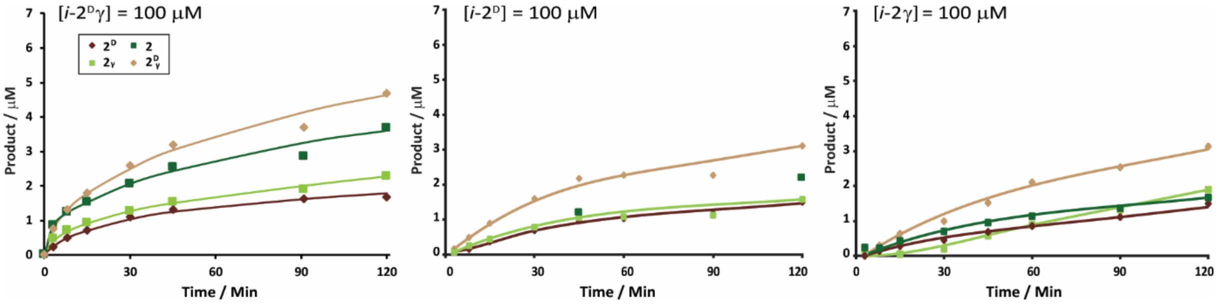

Supplementary figure 9. Time dependent product formation in a reaction mixture initiated with **E** and **N** in presence of the external templates **i-2^{D}**γ, **i-2^{D}** or **i-2**γ (100±10 μM). Due to overlap in the HPLC elution times of **2** and **i-2^{D}**, we were only able to quantify the ammount of peptide **2** produced in this reaction at later stages.

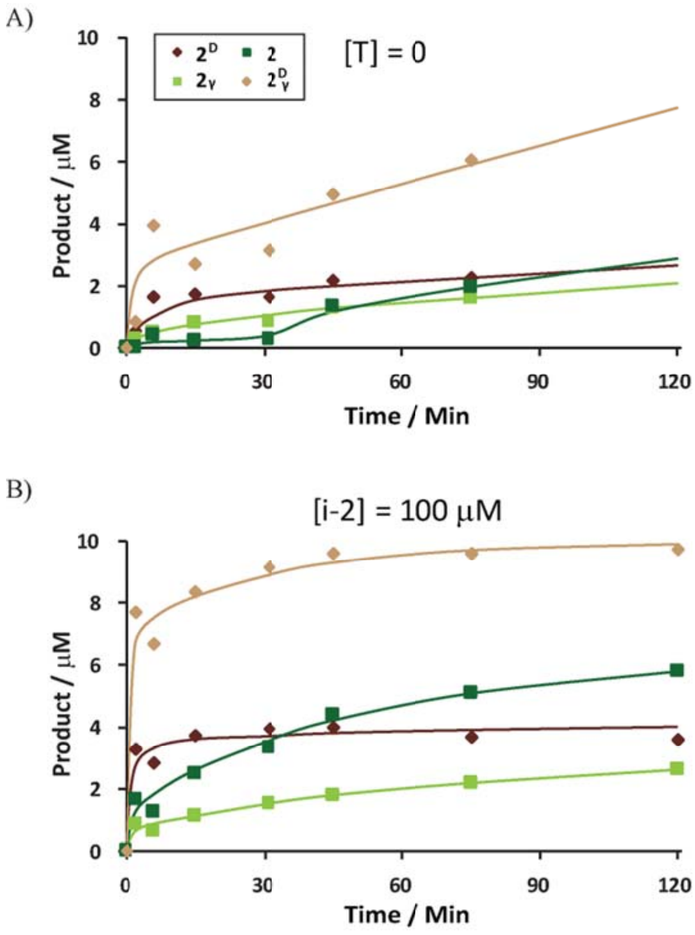

Supplementary figure 10. Time dependent product formation in reaction mixtures initiated with $\mathbf{E^D}$ and **N**. A) template free reaction, and B) in presence of the external template **i-2**.

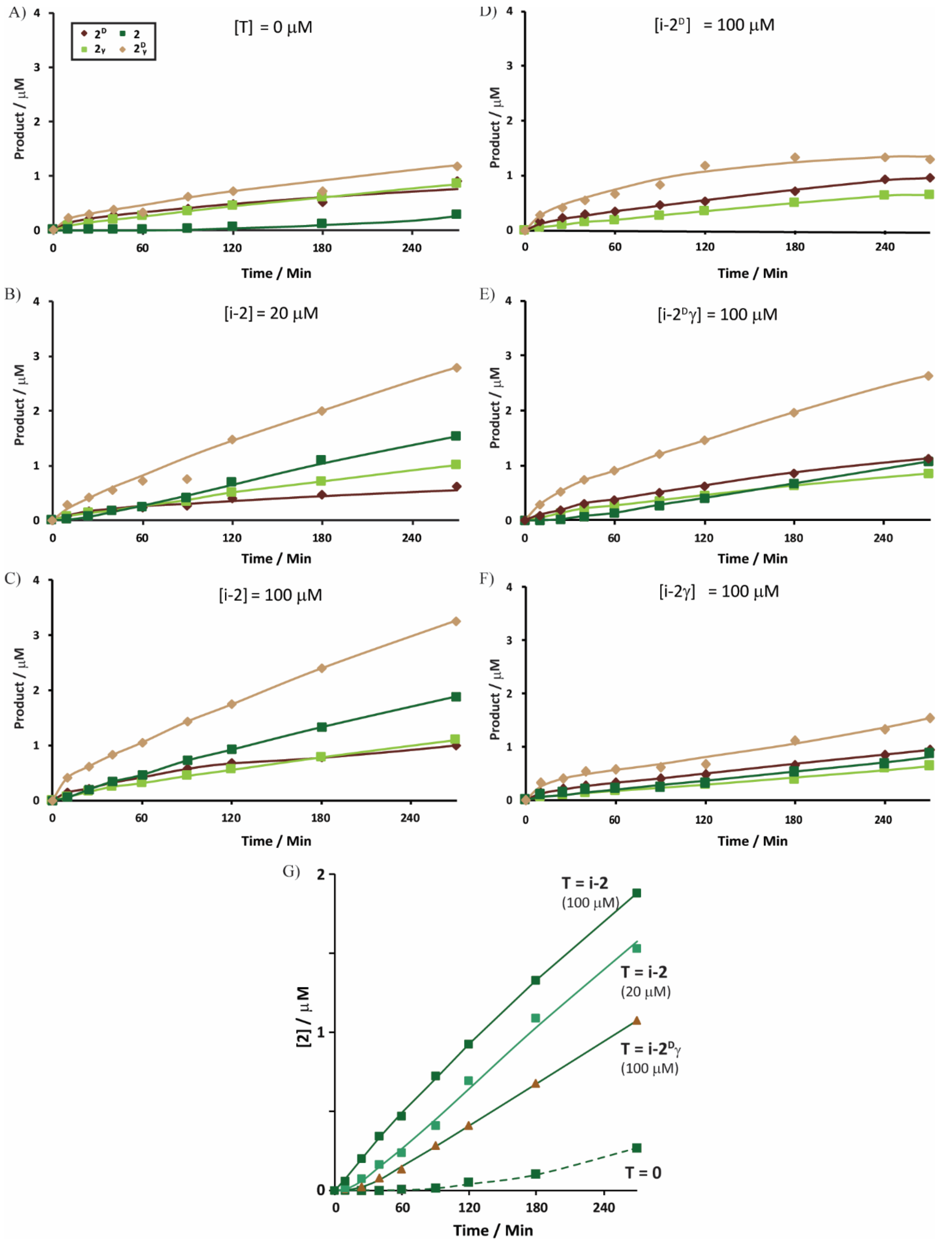

Supplementary figure 11. Time dependent product formation in reaction mixtures initiated with **E**$^{\mathbf{D}}\gamma$ and **N**. A) template-free reaction, B, C) in presence of different ammounts of the external template **i-2**, D) in presence of the external template **i-2**$^{\mathbf{D}}$. Due to overlap in the HPLC elution times of **2** and **i-2**$^{\mathbf{D}}$, we were unable to exactly quantify the ammount of peptide **2** produced in this reaction. E) in presence of external template **i-2**$^{\mathbf{D}}\gamma$ and F) in presence of external template **i-2**γ. G) comparison of the rate of formation of the native peptide **2** under the various reaction conditions.

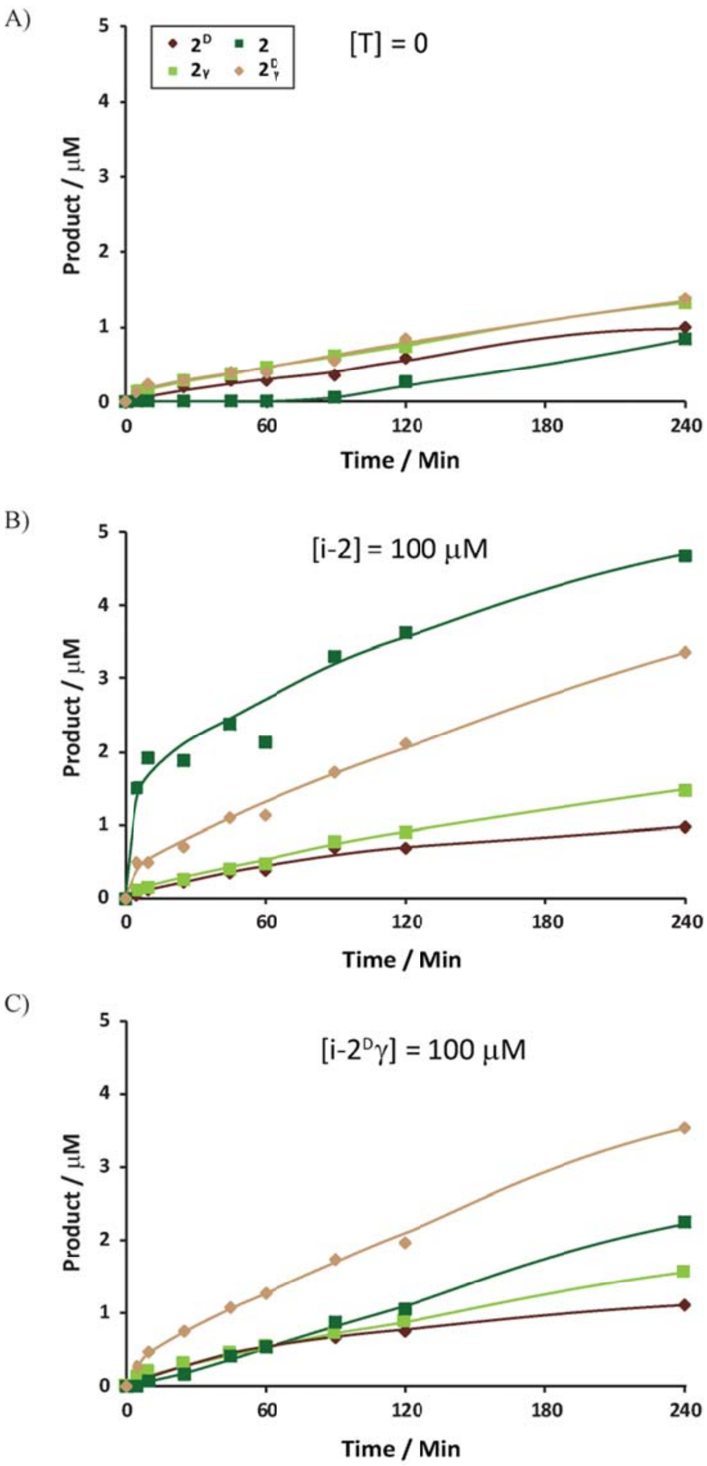

Supplementary figure 12. Time dependent product formation in reaction mixtures in**i**tiated with **E**γ and **N** : A) template free reaction, B) in presence of the external template **i-2** , and C) in presence of the external template **i-2**$^{\mathbf{D}}$γ.

1) Template fibril growth

$$F_i^{\alpha L} + F_1^{\alpha L} \underset{a_{-1}}{\overset{a^{\alpha L}}{\rightleftarrows}} F_{i+1}^{\alpha L}$$

$$F_i^{\alpha D} + F_1^{\alpha D} \underset{a_{-1}}{\overset{a^{\alpha D}}{\rightleftarrows}} F_{i+1}^{\alpha D}$$

$$F_i^{\gamma L} + F_1^{\gamma L} \underset{a_{-1}}{\overset{a^{\gamma L}}{\rightleftarrows}} F_{i+1}^{\gamma L}$$

$$F_i^{\gamma D} + F_1^{\gamma D} \underset{a_{-1}}{\overset{a^{\gamma D}}{\rightleftarrows}} F_{i+1}^{\gamma D}$$

$$(i = 1, \dots, n-1)$$

2) Fibril decomposition

$$F_i^{\alpha L} \overset{d}{\rightarrow} F_{i/2}^{\alpha L} + F_{i/2}^{\alpha L}$$

$$F_i^{\alpha D} \overset{d}{\rightarrow} F_{i/2}^{\alpha D} + F_{i/2}^{\alpha D}$$

$$F_i^{\gamma L} \overset{d}{\rightarrow} F_{i/2}^{\gamma L} + F_{i/2}^{\gamma L}$$

$$F_i^{\gamma D} \overset{d}{\rightarrow} F_{i/2}^{\gamma D} + F_{i/2}^{\gamma D}$$

$$(i = 2m, \dots, n)$$

3) Fibril to tube transition

$$F_i^{\alpha L} \overset{w}{\rightarrow} U_i^{\alpha L}$$

$$F_i^{\alpha D} \overset{w}{\rightarrow} U_i^{\alpha D}$$

$$F_i^{\gamma L} \overset{w}{\rightarrow} U_i^{\gamma L}$$

$$F_i^{\gamma D} \overset{w}{\rightarrow} U_i^{\gamma D}$$

$$(i = m, \dots, n)$$

4) Anhydride formation

$$E^{\alpha L} \underset{k_{-1}}{\overset{k^{\alpha}}{\rightleftarrows}} A^{L} + S$$

$$E^{\alpha D} \underset{k_{-1}}{\overset{k^{\alpha}}{\rightleftarrows}} A^{D} + S$$

$$E^{\gamma L} \underset{k_{-1}}{\overset{k^{\gamma}}{\rightleftarrows}} A^{L} + S$$

$$E^{\gamma D} \underset{k_{-1}}{\overset{k^{\gamma}}{\rightleftarrows}} A^{D} + S$$

5) Anhydride equilibration

$$A^{L} \underset{e_{-1}}{\overset{e}{\rightleftarrows}} A^{D}$$

6) Template free ligation

$$A^{L} + N \overset{g^{\alpha}}{\rightarrow} F_1^{\alpha L}$$

$$A^{D} + N \overset{g^{\alpha}}{\rightarrow} F_1^{\alpha D}$$

$$A^{L} + N \overset{g^{\gamma}}{\rightarrow} F_1^{\gamma L}$$

$$A^{D} + N \overset{g^{\gamma}}{\rightarrow} F_1^{\gamma D}$$

7) Direct ligation

$$E^{\alpha L} + N \overset{g}{\rightarrow} F_1^{\alpha L} + S$$

$$E^{\alpha D} + N \overset{g}{\rightarrow} F_1^{\alpha D} + S$$

$$E^{\gamma L} + N \overset{g}{\rightarrow} F_1^{\gamma L} + S$$

$$E^{\gamma D} + N \overset{g}{\rightarrow} F_1^{\gamma D} + S$$

8) Template assisted ligation

$$A^{L} + N + F_i^{\alpha L} \overset{b^{\alpha L}}{\rightarrow} F_{i+1}^{\alpha L}$$

$$A^{L} + N + F_i^{\gamma L} \overset{b^{\gamma L}}{\rightarrow} F_{i+1}^{\gamma L}$$

$$A^{D} + N + F_i^{\alpha D} \overset{b^{\alpha D}}{\rightarrow} F_{i+1}^{\alpha D}$$

$$A^{D} + N + F_i^{\gamma D} \overset{b^{\gamma D}}{\rightarrow} F_{i+1}^{\gamma D}$$

$$A^{L} + N + F_i^{\alpha L} \overset{c^{\gamma L}}{\rightarrow} F_i^{\alpha L} + F_1^{\gamma L}$$

$$A^{L} + N + F_i^{\gamma L} \overset{c^{\alpha L}}{\rightarrow} F_i^{\gamma L} + F_1^{\alpha L}$$

$$A^{D} + N + F_i^{\alpha D} \overset{c^{\gamma D}}{\rightarrow} F_i^{\alpha D} + F_1^{\gamma D}$$

$$A^{D} + N + F_i^{\gamma D} \overset{c^{\alpha D}}{\rightarrow} F_i^{\gamma D} + F_1^{\alpha D}$$

$$A^{L} + N + F_i^{\alpha D} \overset{c^{\alpha L}}{\rightarrow} F_i^{\alpha D} + F_1^{\alpha L}$$

$$A^{L} + N + F_i^{\gamma D} \overset{c^{\gamma L}}{\rightarrow} F_i^{\gamma D} + F_1^{\gamma L}$$

$$A^{D} + N + F_i^{\alpha L} \overset{c^{\alpha D}}{\rightarrow} F_i^{\alpha L} + F_1^{\alpha D}$$

$$A^{D} + N + F_i^{\gamma L} \overset{c^{\gamma D}}{\rightarrow} F_i^{\gamma L} + F_1^{\gamma D}$$

$$A^{L} + N + F_i^{\alpha D} \overset{c^{\gamma L}}{\rightarrow} F_i^{\alpha D} + F_1^{\gamma L}$$

$$A^{L} + N + F_i^{\gamma D} \overset{c^{\alpha L}}{\rightarrow} F_i^{\gamma D} + F_1^{\alpha L}$$

$$A^{D} + N + F_i^{\alpha L} \overset{c^{\gamma D}}{\rightarrow} F_i^{\alpha L} + F_1^{\gamma D}$$

$$A^{D} + N + F_i^{\gamma L} \overset{c^{\alpha D}}{\rightarrow} F_i^{\gamma L} + F_1^{\alpha D}$$

$$\left[T_{tot}^{\alpha L}\right] = \sum_{i=1}^{n} i\left(\left[F_i^{\alpha L}\right] + \left[U_i^{\alpha L}\right]\right)$$

$$\left[T_{tot}^{\gamma D}\right] = \sum_{i=1}^{n} i\left(\left[F_i^{\gamma D}\right] + \left[U_i^{\gamma D}\right]\right)$$

$$\left[T_{tot}^{\gamma L}\right] = \sum_{i=1}^{n} i\left(\left[F_i^{\gamma L}\right] + \left[U_i^{\gamma L}\right]\right)$$

$$\left[T_{tot}^{\alpha D}\right] = \sum_{i=1}^{n} i\left(\left[F_i^{\gamma D}\right] + \left[U_i^{\gamma D}\right]\right)$$

Supplementary figure 13. Model used for simulating the network analysis of Fig. 5, using the following sets of chemical equations: multiple fibril growth and assembly steps (Eqs. 1-3); production, isomerization and consumption of the anhydrides (Eqs. 4-6); template free (Eqs. 7) and template assisted (Eqs. 8) ligation reactions. Here the specific isomers are named explicitly: the electrophiles **E**, $\mathbf{E^{D}}$, **Eγ** and $\mathbf{E^{D}\gamma}$ are referred to as $E^{\alpha L}$, $E^{\alpha D}$, $E^{\gamma L}$ and $E^{\gamma D}$; the products **2**, $\mathbf{2^{D}}$, **2γ** and $\mathbf{2^{D}\gamma}$ are referred to as $F_i^{\alpha L}$, $F_i^{\alpha D}$, $F_i^{\gamma L}$ and $F_i^{\gamma D}$, the growing fibrils of length i, or as $U^{\alpha L}$, $U^{\alpha D}$, $U^{\gamma L}$ and $U^{\gamma D}$, the tubes that have stopped growing; and the anhydrides **A** and $\mathbf{A^{D}}$ are referred to as A^{L} and A^{D}. N and S are the nucleophile and the released thiol, respectively. The rate constants for each reaction and reverse reaction are also listed explicitly and are grouped according to type; they are given a specific isomer label only when assumed to be isomer dependent. The template assisted ligation reactions are labelled with *b* when the template fibers produce the

respective monomer, and by *c* when the templates catalyse the formation of another type of isomer; for example, $b^{\alpha L}$ is the rate constant when $F_{i+1}^{\alpha L}$ is formed in template assisted ligation by $F_i^{\alpha L}$, and $c^{\alpha L}$ is the rate constant when $F_1^{\alpha L}$ is formed in template assisted ligation by fibril of the other isomers. In all runs presented here we used fibril critical sizes of m = 10 and n = 100.

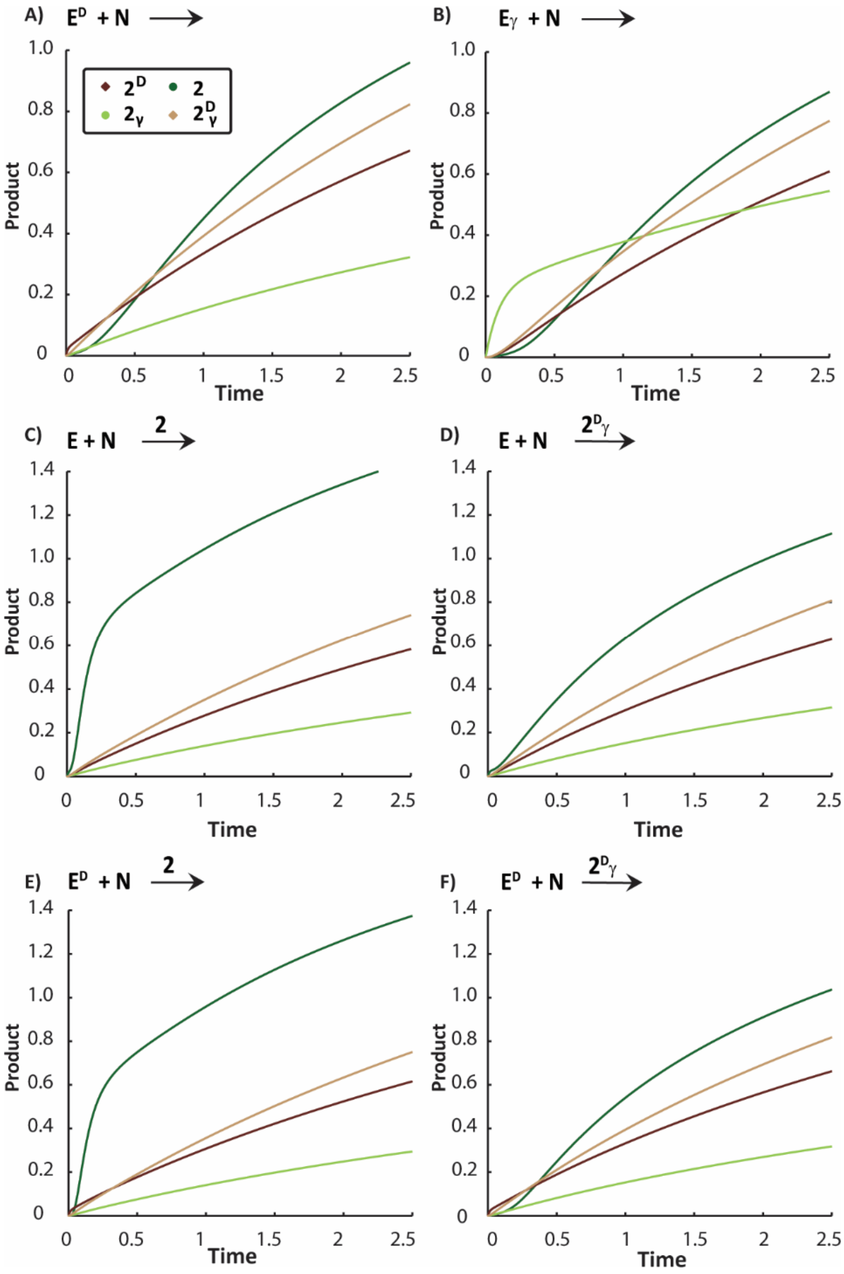

Supplementary figure 14. Additional simulation results based on the mechanism in Figure 5 of the manuscript and the reaction model depicted in Supplementary Figures 13. As in Figure 8 of the manuscript, experimental initial concentrations were assigned for **Ex** and **N** (250 μM each) and reaction constants were applied as specified. a, b) "Error correction" mechanism observed when the reactions were initialized with **N** and $\mathbf{E^D}$ (a) or **E**γ (b). c-f) "Error correction" mechanism observed when the reactions were initialized with **N** and **E** or $\mathbf{E^D}$ and seeded with templates **2** (c,e) or $\mathbf{2^D}$γ (d,f).

Supplementary References

s1. Schmidt, M. W.; Baldridge, K. K.; Boatz, J. A.; Elbert, S. T.; Gordon, M. S.; Jensen, J. H.; Koseki, S.; Matsunaga, N.; Nguyen, K. A.; Su, S. J.; Windus, T. L.; Dupuis, M.; Montgomery, J. A. *J. Comput. Chem.* 1993, **14**, 1347.

s2. http:/www.msg.ameslab.gov/GAMESS/GAMESS.html.
